# Chemical fractionation and mobility of traffic-related elements in road environments

**DOI:** 10.1007/s10653-017-9983-9

**Published:** 2017-05-27

**Authors:** Ewa Adamiec

**Affiliations:** AGH University of Science and Technology, 30 Mickiewicza Av., 30-059 Kraków, Poland

**Keywords:** Brake lining, Non-exhaust emission, Oxidation stress, Heavy metals, BCR, Road dust

## Abstract

Due to considerable progress in exhaust control emission technology and extensive regulatory work regarding this issue, non-exhaust sources of air pollution have become a growing concern. This research involved studying three types of road environment samples such as road dust, sludge from storm drains and roadside soil collected from heavily congested and polluted cities in Poland (Krakow, Warszawa, Opole and Wroclaw). Particles below 20 µm were examined since it was previously estimated that this fine fraction of road dust is polluted mostly by metals derived from non-exhaust sources of pollution such as brake linings wear. Chemical analysis of all samples was combined with a fractionation study using BCR protocol. It was concluded that the finest fractions of road environment samples were significantly contaminated with all of the investigated metals, in particular with Zn, Cu, both well-known key tracers of brake and tire wear. In Warszawa, the pollution index for Zn was on average 15–18 times the background value, in Krakow 12 times, in Wroclaw 8–12 times and in Opole 6–9 times the background value. The pollution index for Cu was on average 6–14 times the background in Warszawa, 7–8 times in Krakow, 4–6 times in Wroclaw and in Opole 5 times the background value. Fractionation study revealed that mobility of examined metals decreases in that order: Zn (43–62%) > Cd (25–42%) > Ni (6–16%) > Cu (3–14%) > Pb (1–8%). It should, however, be noted that metals even when not mobile in the environment can become a serious health concern when ingested or inhaled.

## Introduction

Air pollution is regarded to be the biggest environmental risk factor responsible for premature deaths around the world (EEA 2016). As indicated by European Environmental Agency (EEA 2014), 64% to even 92% of EU urban population is exposed to heavy loads of PM10 and PM2.5 particles. As a result, life expectancy of Europeans is reduced, on average, by about 8.6 months (WHO 2013) or even up to 22 months in the most polluted cities (Aphekom 2011). However, even despite ongoing fight for better air quality, the vast majority of population in urban areas is exposed to air which does not comply with any WHO Air Quality Guidelines.

Road vehicle emissions include exhaust and non-exhaust emissions, both strongly contributing to the overall air pollution problem. Extensive regulatory work regarding exhaust emission and technological improvements introduced by automobile industry have limited the percentage contribution of vehicle tailpipe emissions to total ambient PM concentrations (Gehrig et al. 2004; Thorpe and Harrison 2008; Mathissen et al. 2011; Pant and Harrison 2013), while non-exhaust particle emissions still remain high. As indicated by Dahl et al. (2006) and Kumar et al. (2013), even with zero tail pipe emissions, traffic will continue to contribute fine and ultra-fine particles through non-exhaust emissions. Moreover, Rexeis and Hausberger (2009) estimated that, by the end of the decade, nearly 90% of the total emissions from road traffic will come from non-exhaust sources. Since most studies on toxicity and health consequences of roadside PM are still focusing on diesel exhaust particles (DEPs), particles from the non-exhaust emission (sourced from wearable parts of vehicles) have not yet been so well recognized nor documented (van der Gon et al. 2013). Substantial amount of road dust could also be resuspended (50% according to Garg et al. 2000; Bukowiecki et al. 2010; Kukutschová et al. 2011) and up to 50% of brake wear dust can enter the river system (Garg et al. 2000; Pratt and Lottermoser 2007; Bukowiecki et al. 2009). Moreover, health effects of non-exhaust emission particles are ignored at the regulatory level, despite their links with cardiopulmonary toxicity (Riediker et al. 2004a, b; Gottipolu et al. 2008; Gasser et al. 2009; Mantecca et al. 2009). Wear of brake pads is considered to be the primary source of non-exhaust emission, contributing to as much as 55 mass% in PM10 emission (Harrison et al. 2012) or approximately 21% of the total emissions from road traffic (Bukowiecki et al. 2009; Gasser et al. 2009; Lawrence et al. 2013). Due to variety of materials used in the production of brake linings, the existing knowledge on the impact of new materials on both health and environment is not sufficient. Nowadays an exact amount of materials used in the braking systems is difficult to estimate; however, Chan and Stachowiak (2004) have described the most commonly used brake pad components. Since modern brakes are composed of various compounds, they differ significantly in chemical composition from the first-generation brake materials (Kukutschová et al. 2011). Table 1 contains the compilation of the most popular brake materials and additives used in brake systems.

**Table 1:** Components of brake friction materials. *Source*: Eriksson et al. (1999), Gudmand-Hoyer et al. (1999), Eriksson and Jacobson (2000) and Chan and Stachowiak (2004)

Function	Component and characteristics
Reinforcing fibres	*Glass*—physically strong when bounded together with resinous binders, they exhibit thermal resilience *Metallic* (steel, brass and copper)—in form of chips or granules, mixed with Zn minimizes corrosion; oxidized or phosphatized fibres improve toughness and strength *Aramid*—relatively soft, very light, excellent thermal stability, very good stiffness, superior wear resistance as well as anti-fade properties compared to asbestos, because carcinogenic characteristics should not be considered as asbestos replacement *Ceramic* (potassium titanate)—thermally resilient, very hard, good wear resistance, high thermal stability but light material *Sepiolite* (hydrated magnesium silicate mineral)—excellent sorption properties, stable in high-temperature (even above 1000 °C), because carcinogenic characteristics should not be considered as asbestos replacement
Inorganic fillers	*Barium sulphate*—increases density and wear resistance; stable at high temperature *Calcium carbonate*—cheaper alternative to barium sulphate, but not stable at high temperatures. It imparts heat stability to the friction material, thereby improving brake fade properties *Mica*—suppresses low-frequency brake noise, causes interlayer splitting in friction material *Vermiculite*—exfoliates rapidly into flakes at approximately 800 °C; suppress noises generated during braking *Alkali metal titanates* (e.g. potassium and sodium titanates)—promotes stability of the friction coefficient *Molybdenum trioxide*—prevents thermal fade and cracking of friction lining under high-temperature conditions
Organic fillers	*Cashew dust and rubber*—reduces brake noises due to its superior viscoelastic characteristics. Low thermal conductivity prevents heat from transmitting to the backing plate of the brake friction material. Cashew particles are also able to reduce fluctuations in friction coefficients, especially at elevated temperatures
Binders	*Phenolic resin*—cheap brake friction material acts as a matrix for binding together different substrates; brittle, low impact resistance, highly toxic, decomposes at relatively low temperature, (450 °C) *Silicone*-*modified Phenolic resin*—better impact resistance than pure phenolic resin; better heat and chemical resistance than pure phenolic resin; enhanced water repellency *Epoxy*-*modified Phenolic resin*—better heat resistance than pure phenolic resin. A pure epoxy resin is unable to withstand high temperatures (above 260 °C) *COPNA resin* (condensed polynuclear aromatic resin)—its heat resistance is not significantly better than phenolic resin as it decomposes at approximately the same temperature (400–500 °C) *Cyanate ester resins*—high heat resistance, chemically inert, vibration dampener Brittle, low-impact resistance. Brake pads with cyanate ester resins maintain their friction coefficients at elevated temperatures above 350 °C and good adhesion *Thermoplastic polyamide resin*—abrasion resistant; does not exhibit thermal fade, thermal conductivity three times lower than phenolic resin
Frictional additives	*Graphite*—can be of natural or synthetic origin, in form of flakes or powder. In the flake form it improves lubrication properties, while in powder form is able to dissipate heat generate during braking more effectively *Metal sulphides* (PbS, SnS_2_, CuS, Sb_2_S_3_) have good lubricating properties, with lower conductivities in comparison to graphite. Toxicity of certain compounds such as lead and antimony sulphides is a disadvantage. Therefore, relatively safer alternatives such as tin and copper sulphides would be ideal as lubricants *Aluminium oxide*—hydrated form added as polishing and wear resistance agent. It is suitable as reinforcing fibres used in brake pads and discs since it has high strength-weight ratio than metallic fibres *Magnetite* (Fe_3_O_4_) improves cold friction and is a mild abrasive polishing component *Haematite* (Fe_2_O_3_) is a mild abrasive polishing component *Zinc oxide* (ZnO) lubricates and polish drum *Chromium oxide* (Cr_2_O_3_) raises friction *Lead oxide* (PbO)—friction modifier

As reported by many authors, e.g. Abu-Allaban et al. (2003), Tervahattu et al. (2006), Thorpe et al. (2007), Kam et al. (2012), non-exhaust emissions contribute mainly to the coarse fraction of PM (2.5–10 µm) and exhaust emissions contribute predominantly to fine PM of diameter <2.5 µm. However, authors such as Vu et al. (2015), Grigoratos and Martini (2015) and Adamiec et al. (2016) report that fraction <20 µm is the best indicator of non-exhaust traffic emission, since 90% of all metals originated from brake pads belong to this particular fraction. The fine fraction of road dust (<20 µm) should, therefore, be of special concern, since when inhaled or ingested under acidic pH, it can cause a serious threat to human health and life. Kennedy and Hinds (2002) for instance conclude that particles of up to 100 µm are inhalable (both via mouth and nose) and could lodge/deposit in the upper part of the respiratory tract. There are only scarce studies on transformation, mobility and speciation of non-exhaust traffic-related metals bound in the solid phase.

There is a considerable body of research on roadside soil (e.g. Carrero et al. 2013). For example, Werkenthin et al. (2014) present a very interesting survey of studies on metals in European roadside soils. Interestingly, there are fewer studies on road dust and only sporadic projects on sludge. Filgueiras et al. (2002) reviewed 400 extraction protocols for metal fractionation in environmental samples, and only 4 studies were conducted on road dust samples. Moreover, Świetlik et al. (2015) conclude that based on Scopus database of over 1500 abstracts and articles containing keyword “road dust” only approximately 5% of studies involved solid-phase speciation of heavy metals in road dust. Therefore, there is a substantial need for new studies on metal fractionation in road environments since it provides useful information on mobility and bioavailability of contaminants. Metals in road environments bound in various fractions such as carbonates, sulphides, organic matter, iron, aluminium and manganese oxyhydroxides as well as phyllosilicate minerals, and they undergo various processes such as ion exchange, sorption and/or adsorption as well as precipitation and/or co-precipitation. Bioavailability of heavy metals depends greatly on the characteristics of the particle surface or bond strength. Changes in chemical or physical conditions such as pH, temperature, redox potential, organic matter decomposition, leaching, ion exchange processes and microbial activity in the environment all influence the release of toxic metals into environment. Fractionation with the use of BCR protocol is a useful tool to distinguish between the anthropogenic and geochemical sources of metals in solid samples. It also provides essential information on the mobility and bioavailability of metals, transport mechanisms and, consequently, the impact those metals have on the environment and human health.

The aim of the study was to investigate various samples taken from road environment (such as road dust, sludge from storm drains and pavement dust), which are influenced mostly by non-exhaust emission and are located in close vicinity to urban roads in four biggest cities in Poland (Krakow, Warszawa, Wroclaw and Opole). Chemical analysis followed by the fractionation study using BCR protocol was conducted on the fraction <20 µm since this fraction has rarely been examined by researchers despite the fact that it should be regarded to be the best indicator of non-exhaust traffic emission.

## Materials and methods

### Sampling area

**Fig. 1:**
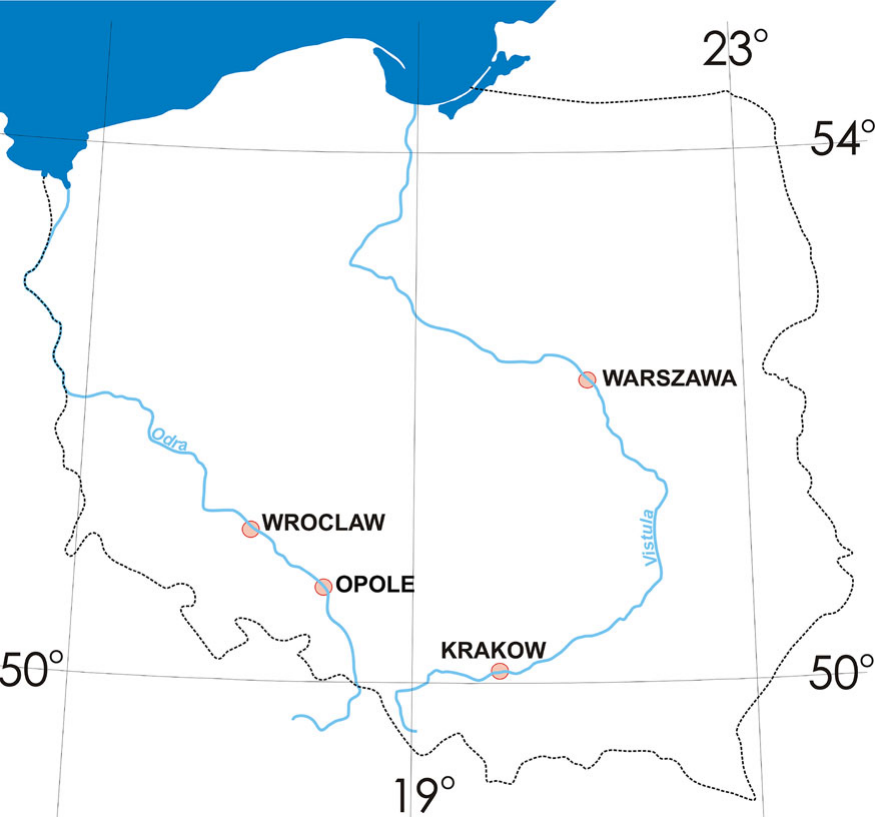
Sampling areas

 The sampling points (Table 2; Fig. 1) have been specifically selected to minimize the impact of other sources of contamination. Samples were collected in the period from May 2015 to December 2015 during summer and autumn sampling campaigns. Total of 148 samples from Krakow, Warszawa, Wroclaw and Opole were examined. Out of 12 sampling points, 8 are heavily affected by traffic and 4 are considered to be free of traffic influence. In each of those 12 research areas, samples of road sediment, sludge form storm drains and roadside topsoil (from 20 cm depth) were collected in summer and autumn sampling campaigns in 2015. Samples of road dust, including field duplicates, were collected in situ using a vacuum cleaner specifically modified for collecting road dust; some wet samples were swept with a brush from the road (rectangle 4 m × 2 m). Sludge was sampled from storm drains in the amounts of about 1000 mg. Roadside topsoil was collected from square sections of 1 m × 1 m, at 20 cm depth (about 10 m away from the bridge).

**Table 2:**
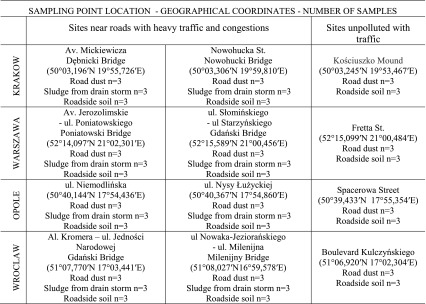
Sampling point location

### Methods

 Metals were extracted from road dust, roadside topsoil and sludge from storm drains with *aqua regia* using microwave oven digestion protocol 3050B (EPA 1996). Fractionation study was conducted on 12 preselected samples according to BCR protocol (Ure et al. 1993). Table 3 presents reagents for sequential extraction procedure.

**Table 3:** Sequential extraction procedure according to BCR protocol

Step	Fraction	Targets phases	BCR protocol (Ure et al. 1993)
1	Exchangeable, water and acid soluble	Soluble species, carbonates, cation exchangeable sites	0.11 mol/L acetic acid
2	Reducible	Fe and Mn oxyhydroxides	0.1 mol/L hydroxylammonium chloride, pH 2
3	Oxidizable	Organic matter and sulphides	Hydrogen peroxide followed by 1.0 mol/L ammonium acetate, pH 2
4^a^	Residual	Remaining, non-silicate bound metals	*Aqua regia* digestion

^a^The residual extraction step is not included in original procedure, but it can be useful for quality control, since sum of steps 1–4 can be compared with digestion protocol 3050B (EPA 1996; Mossop and Davidson 2003)

The concentrations of Cd, Cu, Ni, Pb and Zn were analysed using inductively coupled plasma-mass spectroetry (ICP-MS) (ELAN 6100; PerkinElmer) according to US EPA method 6020A (EPA 1998). The concentrations of metals in road dust were then compared with the geochemical background based on Turekian and Wedephol (1961), since there are no geochemical background values determined for Polish soils in the fraction <20 μm. World average shale values (ASV), considered to be the reference for the fraction of 20 µm, were as follows: for Cd (0.3 mg/kg), Cu (45 mg/kg), Ni (68 mg/kg), Pb (20 mg/kg) and Zn (95 mg/kg). Furthermore, I geo index was calculated according to Müller (1969):

1 $$I_{\text{geo}} = \log_{2 } \cdot \left( {\frac{{C_{\text{n}} }}{{1.5 \cdot B_{\text{n}} }}} \right) ,$$

where *C*_n_ is the concentration of element **n** and *B*_n_ is the geochemical background.

### Data quality

To obtain unambiguous and unbiased ICP-MS results, elements were also measured using inductively coupled plasma-optical emission spectroscopy (ICP-OES) (OPTIMA 7300DV; PerkinElmer), atomic absorption spectroscopy (AAS) (F-AAS Thermo Scientific IC 3500) according to US EPA method 7000 in the Laboratory of Trace Analyses at the Faculty of Geology, Geophysics and Environmental Protection, AGH University of Science and Technology. To estimate the accuracies and biases of the analytical methods, reagent blanks and certified international reference materials BCR 701 (lake sediment), BCR 723 (road dust), ERM-CZ120 (fine dust PM_10_-like) (EU JRC IRMM), METRANAL™32 (light sandy soil, grain size <100 µm) (Analytika Czech Republic) as well as SRM 1848a (urban particulate matter) (NIST) were used to ensure that the obtained results met the required criteria. Analyses of the reference materials verified and confirmed the quality of the results. Analytical bias was statistically insignificant (*p* = 0.05), and the precisions of AAS and ICP-MS systems were satisfactory, which was verified by six different solution injections. Rh was used as an internal standard. Using ICP-MS, element correction equations were used for each element to minimize the impact of interferences.

## Results and discussion

Concentrations of Cu and Zn, the well-known key tracers of non-exhaust brake and tire wear, were highly elevated in fine fractions of all types of road environment samples in the examined cities. In Warszawa, the pollution index (Chen et al. 2005; Radziemska and Fronczyk 2015) calculated for Zn was on average 15–18 times the background value, in Krakow 12 times, in Wroclaw 8–12 times and in Opole 6–9 times the background value. Furthermore, the pollution index for Cu was found to be on average 6–14 times the background value in Warszawa road environment, 7–8 times in Krakow, 4–6 times in Wroclaw and 5 times the background value in Opole. Road dust and sludge from storm drains were the most contaminated of all road environment samples in each of the cities; however, Krakow and Warszawa were especially contaminated with Cu and Zn due to heavy traffic congestions as well as poor infrastructure.

The highest concentrations of Cd in all road environment samples were found in Krakow and Wroclaw. Similar results were obtained for the concentration of Pb, which was the highest in sludge from storm drains and road dust also in Krakow and Wroclaw. Statistical parameters of the examined road environment samples are depicted in Table 4.

**Table 4:** Statistical parameters of road environmental samples

Element (mg/kg)	Cd	Cu	Pb	Ni	Zn
Kraków					
Road dust (*n* = 6)					
Min–max	1.65–2.56	145–484	98.7–285	37.2–49.9	600–1230
Mean/median	2.29/2.40	363/384	196/178	44.0/43.9	1043/1148
Sludge (*n* = 6)					
Min–max	2.85–3.28	276–334	177–213	40.3–46.6	1049–1302
Mean/median	3.01/3.01	305/308	197/198	43.7/44.3	1177/1174
Topsoil (*n* = 6)					
Min–max	2.77–6.04	89.0–189	146/445	39.3–61.4	881–1844
Mean/median	3.83/3.96	155/160	218/185	51.3/55.2	1292/1255
Warszawa					
Road dust (*n* = 6)					
Min–max	0.863–2.09	136–1055	214–258	34.5–88.1	651–2170
Mean/median	0.966/0.976	606/649	241/245	64.8/72.2	1426/1467
Sludge (*n* = 6)					
Min–max	1.03–6.42	130–970	145–363	25.5–76.8	799–4367
Mean/median	3.21/2.65	580/612	255/272	58.9/63.5	2214/1804
Topsoil (*n* = 6)					
Min–max	0.595–1.34	188–331	105–348	37.9–45.4	760–874
Mean/median	0.943/0.917	262/264	214/220	42.9/42.3	801/787
Wrocław					
Road dust (*n* = 6)					
Min–max	1.07–5.07	70.0–295	101–210	55.8–284	515–1851
Mean/median	1.97/1.58	164/165	161/163	106/76.5	1017/814
Sludge (*n* = 6)					
Min–max	1.24–3.42	257–397	182–301	92.5–146	819–2049
Mean/median	2.25/2.16	313/297	230/219	115/10	1354/1275
Topsoil (*n* = 6)					
Min–max	1.11–3.02	61.0–84.5	118–158	46.7–56	450–1131
Mean/median	2.02/1.98	71.4/70.2	135/132	50.7/50.0	772/754
Opole					
Road dust (*n* = 6)					
Min–max	0.557–1.06	114–312	33.2–94.6	107–299	446–917
Mean/median	0.848/0.889	212/210	64.1/64.4	203/203	680/678
Sludge (*n* = 6)					
Min–max	1.3–2.0	129–817	56.9–190	109–567	779–1757
Mean/median	1.48/1.35	241/231	96.2/75.4	237/141	1127/994
Topsoil (*n* = 6)					
Min–max	1.06–1.81	58.2–106	44.5–56.2	61.1–340	689–888
Mean/median	1.43/1.43	81.9/82.1	50.3/50.3	201/202	786/784

Concentration of metals in the studied road dust was then used to calculate I geo accumulation index according to Müller (1969), and the results are depicted in Table 5. Based on I geo classification, it can be concluded that in Krakow, Warszawa and Wroclaw, due to heavy congested traffic, all types of road environment samples, i.e. road dust, sludge from storm drains and surrounding soil, have been classified mainly as class 3 (moderately to strongly polluted) and class 4 (strongly polluted) with respect to sludge from Warszawa. No elevated concentration of Ni was found in any of the road environment samples.

**Table 5:** Statistical parameters of reference samples unpolluted with traffic

Element (mg/kg)	Cd	Cu	Pb	Ni	Zn
Kraków					
Road dust (*n* = 3)					
Min–max	0.679–0.988	85.4–127	67.5–98.2	35.1–42.8	209–371
Mean/median	0.850/0.882	106/107	80.9/77.1	39.4/40.2	301/322
Topsoil (*n* = 3)					
Min–max	0.477–0.804	79.0–122	54.3–89.3	32.3–44.4	162–289
Mean/median	0.616/0.568	102/102	70.1/76.6	38.7/39.3	218/202
Warszawa					
Road dust (*n* = 3)					
Min–max	0.675–1.09	88.1–148	98.3–168	29.5–45.4	161–370
Mean/median	0.880/0.876	113/102	136/143	36.5/34.5	278/302
Topsoil (*n* = 3)					
Min–max	0.395–0.665	67.5–110	87.3–112	28.8–50.3	107–274
Mean/median	0.574/0.663	88.3/87.4	99.2/98.2	37.8/34.4	193/198
Wrocław					
Road dust (*n* = 3)					
Min–max	0.707–1.77	54.3–69.8	87.4–116	54.0–87.6	167–334
Mean/median	1.25/1.27	60.1/56.3	101/101	76.2/87.0	241/221
Topsoil (*n* = 3)					
Min–max	0.591–1.21	39.2–56.8	76.2–108	43.7–49.3	146–309
Mean/median	0.930/0.988	50.1–54.2	90.5/87.3	46.1/45.3	218/198
Opole					
Road dust (*n* = 3)					
Min–max	0.321–0.744	45.5–87.1	54.2–68.6	59.3–93.4	132–290
Mean/median	0.654/0.744	66.2/65.9	60.5/58.8	76.3/76.3	208/202
Topsoil (*n* = 3)					
Min–max	0.381–1.01	34.4–65.9	38.4–54.2	45.4–102	119–276
Mean/median	0.759/0.886	47.9/43.5	46.0/45.5	75.2/78.1	192/180

**Fig. 2:**
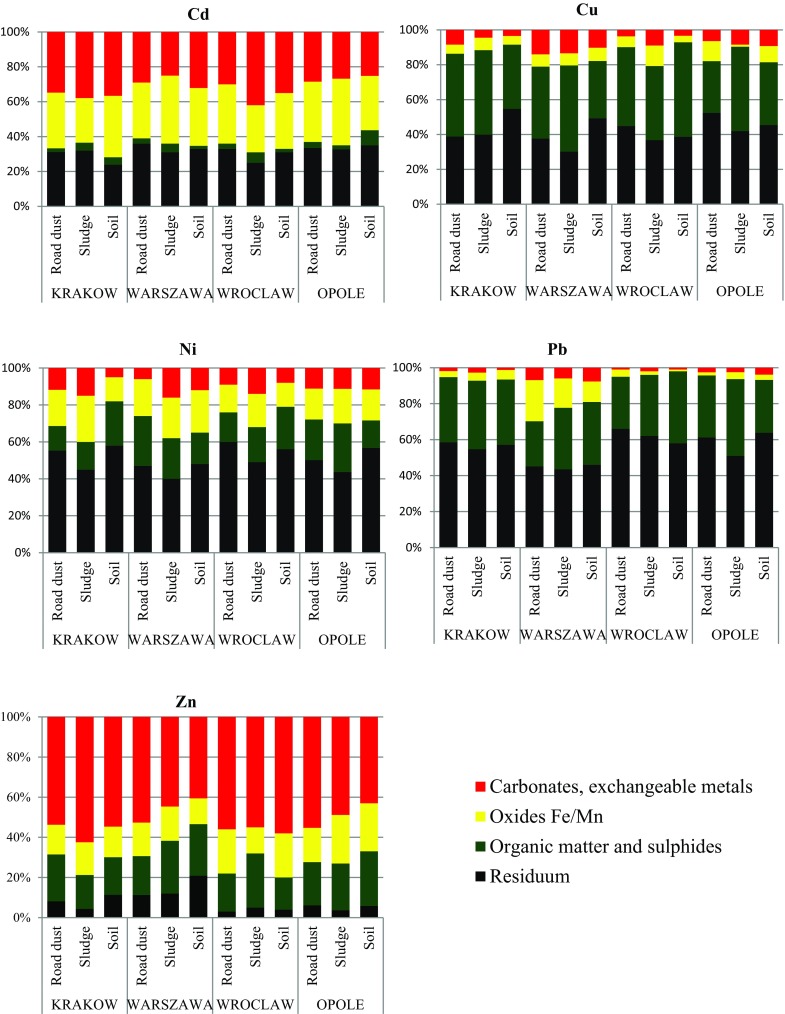
Chemical fractionation of Cd, Cu, Ni, Pb and Zn in grain size <20 µm of road environment samples

Fractionation studies are conducted on the selected samples and depicted in Fig. 2. Table 6 reveals that Zn was the most mobile and bioavailable of all investigated metals; on average, 41–62% of Zn was bound with carbonates and/or exchangeable metal fractions. This heavy metal was also the most abundant in road environment sediment and sludge and came mostly from tire wear. Its high concentrations resulted from the addition of ZnO and ZnS to the tire during vulcanization. Zn and ZnO were associated mainly with carbonates and exchangeable fractions; on average, 54% of Zn was found in road dust, 53% in sludge and 49% in the surrounding soil. Moreover, ZnS and Zn^2+^ are adsorbed on Fe(III) hydroxides on average 18% (in dust and sludge) and 19% in soil. The results are in line with the findings of Świetlik et al. 2015, who estimated that 54% of Zn in road dust collected from Falencice and Jedlińsk E77 expressway near Warszawa, Poland, was predominantly bound with weak acid fraction. Additionally, high mobility was found for Cd, associated significantly (25–42%) with easily leachable carbonates and exchangeable fractions. This specific metal can cause extreme threat to the environment, especially in Warszawa, where its concentration was found to be as high as 6.42 mg/kg in sludge from storm drains, exceeding the background levels 21 times.

**Table 6:**
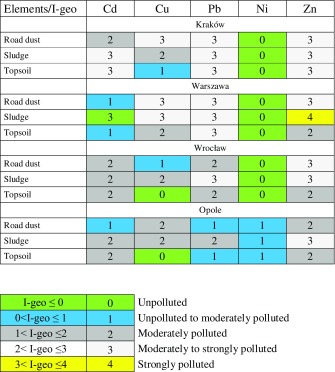
I geo index in the fraction <20 µm of road environment samples

**Table 7:** Mobility and bioavailability of metals in the fractions

*Road dust*	
Carbonates, exchangeable metals	Zn > Cd > Cu ≈ Ni > Pb
Oxides Fe/Mn	Cd > Zn ≈ Ni > Pb > Cu
Organic matter and sulphides	Cu > Pb > Zn > Ni > Cd
Residual	Pb > Ni > Cu > Cd > Zn
*Sludge*	
Carbonates, exchangeable metals	Zn > Cd > Ni > Cu > Pb
Oxides Fe/Mn	Cd > Ni > Zn > Pb > Cu
Organic matter and sulphides	Cu > Pb > Zn > Ni > Cd
Residual	Pb > Ni > Cu > Cd > Zn
*Soil*	
Carbonates, exchangeable metals	Zn > Cd > Ni > Cu > Pb
Oxides Fe/Mn	Cd > Zn > Ni > Pb > Cu
Organic matter and sulphides	Cu > Pb > Zn > Ni > Cd
Residual	Pb > Ni > Cu > Zn > Cd

No significant environmental risk was associated with Ni or Cu; these metals, however, when ingested can be extremely toxic and even lethal. As estimated by Zhou et al. (2015), exposure dose of Pb, Cd, Cu sourced from road dust by ingestion is two orders of magnitude higher than the corresponding dermal dose and up to four orders of magnitude higher than the inhalation exposure. Copper in all samples is bound in 34.6–54.3% with organic matter and sulphites but also with the residuum (30–52%). Total concentration of Ni is rather low in all road environment samples; approximately 90% is bound with stable fractions.

Total concentration of lead is rather high in all examined samples, since this element is an important component of bearing alloys and was used commonly as a material for wheel balancing weight now replaced by zinc weights. Additionally, it should be noted that Pb is a very persistent element. Its elevated concentrations in urban dust could be a consequence of common use of PbO_4_ as a gasoline additive in Poland up to March 2005. Despite high lead concentrations, most of Pb is in bound with stable fractions and only negligible percentage is mobile. In the road environment samples derived from Krakow, Wroclaw and Opole lead is bound approximately 1–2% with carbonates and exchangeable fractions and up to 7.6% in soil in Warszawa.

## Conclusion

The finest fractions of road dust, mixed sludge and sediment from storm drains as well as soil from the vicinity of roads (up to <20 µm) were significantly contaminated with all of the investigated metals, in particular with Zn, Cu, Pb and Cd. Since copper and zinc are well-recognized key tracers of non-exhaust emission, their high concentrations confirm that brake and tire wear highly contributes to road environmental samples contamination with heavy metals. More specifically, it can be concluded that sludge and sediments from storm drains are slightly more contaminated than road dust and soil. Fractionation study revealed that mobility of the examined metals decreases in the following order: Zn (43–62%) > Cd (25–42%) > Ni (6–16%) > Cu (3–14%) > Pb (1–8%) (Table 7). It should be noted, however, that metals (even if not mobile in the environment) can cause serious health concerns when ingested or inhaled. Therefore, metals in road environment samples should also be regarded as a secondary pollution source. Extremely high concentrations of metals in sludge from storm drains in Krakow and Warszawa could potentially pollute the Vistula River, which enters the Baltic Sea, while in Wroclaw and Opole traffic-related metals could enter the Oder River on the border with Germany.
